# Understanding BRCA2 Function as a Tumor Suppressor Based on Domain-Specific Activities in DNA Damage Responses

**DOI:** 10.3390/genes12071034

**Published:** 2021-07-02

**Authors:** Paul R. Andreassen, Joonbae Seo, Constanze Wiek, Helmut Hanenberg

**Affiliations:** 1 Division of Experimental Hematology and Cancer Biology, Cancer and Blood Diseases Institute, Cincinnati Children’s Hospital Medical Center, Cincinnati, OH 45229, USA; JoonBae.Seo@cchmc.org; 2 Department of Pediatrics, University of Cincinnati College of Medicine, Cincinnati, OH 45229, USA; 3 Department of Otorhinolaryngology and Head/Neck Surgery, Heinrich Heine University, 40225 Düsseldorf, Germany; constanze.wiek@med.uni-duesseldorf.de (C.W.); Helmut.Hanenberg@uk-essen.de (H.H.); 4 Department of Pediatrics III, Children’s Hospital, University of Duisburg-Essen, 45122 Essen, Germany

**Keywords:** BRCA2, tumor suppressor, DNA damage responses, DNA repair, homologous recombination, G2 checkpoint, DNA binding, variants of uncertain significance

## Abstract

*BRCA2* is an essential genome stability gene that has various functions in cells, including roles in homologous recombination, G2 checkpoint control, protection of stalled replication forks, and promotion of cellular resistance to numerous types of DNA damage. Heterozygous mutation of *BRCA2* is associated with an increased risk of developing cancers of the breast, ovaries, pancreas, and other sites, thus *BRCA2* acts as a classic tumor suppressor gene. However, understanding *BRCA2* function as a tumor suppressor is severely limited by the fact that ~70% of the encoded protein has not been tested or assigned a function in the cellular DNA damage response. Remarkably, even the specific role(s) of many known domains in BRCA2 are not well characterized, predominantly because stable expression of the very large BRCA2 protein in cells, for experimental purposes, is challenging. Here, we review what is known about these domains and the assay systems that are available to study the cellular roles of BRCA2 domains in DNA damage responses. We also list criteria for better testing systems because, ultimately, functional assays for assessing the impact of germline and acquired mutations identified in genetic screens are important for guiding cancer prevention measures and for tailored cancer treatments.

## 1. Introduction

*BRCA2* is an essential genome stability gene on chromosome 13 that has a key role in DNA repair mediated by homologous recombination (HR), both in response to DNA double-strand breaks (DSBs) and DNA interstrand crosslinks (ICLs) [1,2,3,4]. As part of its role in mediating HR and maintaining genome stability, the BRCA2 protein regulates the assembly of the RAD51 recombinase into a nucleoprotein filament with single-stranded DNA (ssDNA) following resection of DSBs [1,4]. In this manner, BRCA2 promotes strand invasion and the search for homologous DNA. Following the exposure of cells to DNA damage, BRCA2 is recruited to nuclear foci and is required for the assembly of RAD51 foci [5,6]. Another role for BRCA2 in DNA damage responses is in mediating cellular resistance to a variety of DNA damaging agents. These include ionizing radiation (IR), which induces DSBs, mitomycin C (MMC) and cisplatin that induce ICLs, topoisomerase I inhibitors such as camptothecin, and poly (ADP-ribose) polymerase (PARP) inhibitors [6,7,8,9,10,11]. Further, BRCA2 regulates G2 DNA damage checkpoint arrest in response to IR [12,13]. Aside from DNA repair-related roles of BRCA2 in maintaining genome stability, BRCA2 also has functions related to DNA replication, including stabilizing stalled replication forks and protecting them from degradation of nascent DNA [14,15].

Heterozygous germline mutations in *BRCA2* confer a profound predisposition to breast, ovarian, pancreatic, prostate, fallopian tube, and other types of cancer, including melanoma [16,17,18,19]. Importantly, one functional copy of *BRCA2* is typically sufficient to suppress the development of tumors in heterozygous carriers of germline mutations. However, a subsequent mutation in the remaining allele can drive malignant transformation in somatic cells of these healthy individuals, who experience a very high propensity for tumor formation in their lifetime [20]. Therefore, *BRCA2* is a classic tumor suppressor gene, similar to related tumor suppressors such as *BRCA1* and *PALB2*, which also function in the cellular response to DNA damage and in the maintenance of genetic/genomic stability [4,21]. In support of BRCA2 functioning as a tumor suppressor via a role in DNA repair/maintenance of genetic stability, tumors with mutations in *BRCA2* typically display a type 3 base substitution mutation signature [22,23]. It is noteworthy here that the normal allele of *BRCA2* can also be inactivated in various tumor types by epigenetic DNA modifications, such as promoter hypermethylation [24,25,26,27].

In contrast, biallelic germline mutation of *BRCA2* is linked to the D1 subtype of the rare inherited childhood disorder Fanconi anemia (FA) [11]. FA is defined as a chromosome instability syndrome caused by inactivating germline mutations in at least 22 autosomal or X-chromosomal genes [28,29]. FA is typically associated with congenital anomalies, progressive bone marrow failure, and a predisposition to blood-related cancers and solid tumors [28,29]. Non-malignant cells from FA-D1 patients show genome instability—specifically spontaneous and DNA interstrand crosslink (ICL)-induced—chromosome breaks and radial chromosome formation [11]. FA-D1 patients with biallelic mutation in *BRCA2* develop cancers such as myeloid and lymphatic malignancies, medulloblastoma and Wilms tumor in early childhood, typically in as high as 90% of patients by 7 years of age [30,31]. Not surprisingly, heterozygous carriers of *BRCA2* mutations in FA-D1 kindreds display an elevated risk of breast and other cancers [32]. 

Although progress has been made in identifying functions for BRCA2 in DNA damage responses, much remains to be understood about the function of BRCA2 as a tumor suppressor. For example, the domain structure and function of approximately 70% of BRCA2 is still undefined (see Section 2). Importantly, the specific roles of previously identified domains have been incompletely characterized (see Section 4). There is some evidence that different domains within BRCA2 can have distinct activities [14]. Thus, an accurate comprehension of how BRCA2 functions as a tumor suppressor will likely require defining the specific roles of known, and still unknown, domains throughout the protein. It will also be important to understand how domains in different parts of the protein may cooperate in maintaining genome stability. Knowledge of the functions of domains throughout BRCA2 is also necessary for developing and/or implementing effective cancer prevention measures and specific treatments based upon the mutational status and residual function(s) of *BRCA2* in cells [33,34,35,36,37]. Thus, in this review, we begin by critically evaluating what is currently known about the domain structure of BRCA2 (Section 2). We then survey the models and systems utilized to test the roles of different domains of BRCA2 in DNA damage responses, because they have often imposed constraints on investigations of BRCA2 function (Section 3). What is known about the specific roles of different domains of BRCA2 in DNA damage responses, while limited, is also examined (Section 4). We finish by considering how to better test the function(s) of different domains and how BRCA2 function is affected by patient-derived mutations (Section 5).

## 2. Known Domains within BRCA2

**Figure 1:**
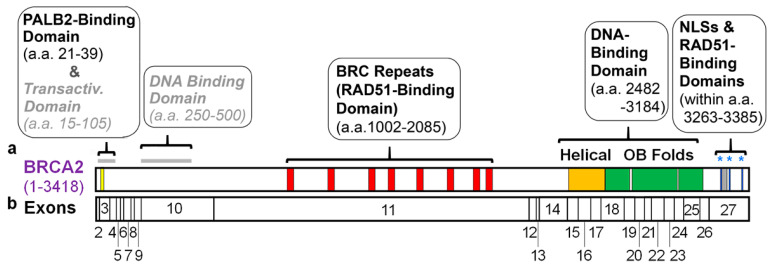
Diagram of the positions of identified domains in human BRCA2 and of the 27 exons that encode this protein. (**a**) At the extreme N-terminus of BRCA2 is a PALB2-binding domain (yellow bar) from a.a. 21 to 39 [38]. The central region of BRCA2 contains a RAD51-binding domain from a.a. 1002 to 2085, including 8 BRC repeats (red bars, BRC1: a.a. 1002–1036, BRC2: a.a. 1212–1246, BRC3: a.a. 1421–1455, BRC4: a.a. 1517–1551, BRC5: a.a. 1664–1698, BRC6: a.a. 1837–1871, BRC7: a.a. 1971–2005, and BRC8: a.a. 2051–2085), and intervening sequences [39,40]. There is also a prominent DNA-binding domain (DBD) at the C-terminus of BRCA2 (C-DBD), spanning a.a. 2482–3184, which includes a helical domain (a.a. 2482–2668, orange bar) and three OB folds (OB1: a.a. 2670–2803, OB2: a.a. 2808–3049, and OB3: a.a. 3055–3184, each shown as a green bar) [41]. There is an additional RAD51-binding domain at the extreme C-terminus of BRCA2 (a.a. 3270–3305, grey bar) [42]. On either side of this motif are three putative nuclear localization signals (NLS) (NLS1: a.a. 3263–3269, NLS2: a.a. 3311–3317 and NLS3: a.a. 3381–3385 [43]) indicated by blue asterisks and bars. A less well characterized N-terminal DBD (N-DBD), present at a.a. 250–500 [44], and a potential transactivation activity revealed via fusion to other factors (present at a.a. 18–105) [45], are shown by a grey line and indicated with grey/italicized text because of uncertainty about their physiological relevance. (**b**) Exons of *BRCA2* are shown relative to the portions of the BRCA2 protein (**a**) they encode. Exons of *BRCA2* by the amino acids they encode: exon 2 (a.a. 1–22), exon 3 (a.a. 23–105), exon 4 (a.a. 106–141), exon 5 (a.a. 142–158), exon 6 (a.a. 159–172), exon 7 (a.a. 173–210), exon 8 (a.a. 211–227), exon 9 (a.a. 228–264), exon 10, (a.a. 265–636), exon 11 (a.a. 637–2280), exon 12 (a.a. 2281–2312), exon 13 (a.a. 2313–2335), exon 14 (a.a. 2336–2478), exon 15 (a.a. 2479–2539), exon 16 (a.a. 2540–2601), exon 17 (a.a. 2602–2658), exon 18 (a.a. 2659–2777), exon 19 (a.a. 2778–2829), exon 20 (a.a. 2830–2877), exon 21 (a.a. 2878–2918), exon 22 (a.a. 2919–2984), exon 23 (a.a. 2985–3039), exon 24 (a.a. 3040–3085), exon 25 (a.a. 3086–3167), exon 26 (a.a. 3168–3216), and exon 27 (a.a. 3217–3418).

Most domains within BRCA2 were initially identified based upon structural elements. We also consider here the domains defined by interactions with PALB2, RAD51, and DSS1, which are key partners of BRCA2. In this section, currently known domains are presented, in order from the N- to the C-terminus of human BRCA2. We discuss how each domain was identified and, as summarized in Figure 1a, where they are located in BRCA2. 

Using antibodies against BRCA2, Xia et al. (2006) identified an orphan protein as one of its major interactors and named it partner-and-localizer-of BRCA2 (PALB2) [46]. They then mapped the PALB2-binding domain to the extreme N-terminus of BRCA2, specifically a conserved region consisting of amino acids (a.a.) 10–40. A subsequent study refined the PALB2-binding region of human BRCA2 to a.a. 21–39 [38]. In vitro pull-down experiments utilizing peptides and/or recombinant fragments of BRCA2 and PALB2 have demonstrated that this is a direct interaction [38,47]. 

Interestingly, a search for homology with known proteins identified a region, in human BRCA2 (a.a. 57–98), with similarity to the c-Jun transcription factor [45]. Via fusion to the lexA and GAL4 DBDs, transactivation by a.a. 18–105 of BRCA2 was demonstrated in yeast and human cells, respectively [45]. The primary activating region was mapped to a.a. 18–60 of BRCA2, while a.a. 60–105 was found as an auxiliary activating region. The specific function, if any, of this transactivating activity in the cellular DNA damage response is unclear (see Section 4.1).

An N-terminal DBD (N-DBD), which was tested only in vitro, was identified on the basis of predicted secondary structure suggestive of a zinc finger-PARP like domain [44]. In particular, blocks from a.a. 267–80 and 314–332 contained predicted DNA-binding residues. In vitro DNA binding assays mapped the N-DBD to a.a. 250–500 of BRCA2, without further dissection. Notably, mutation of C315S, but not C279A and C314S, within the blocks of predicted DNA-binding residues impaired binding to double-stranded DNA (dsDNA) [44]. However, the specific role(s) of the N-DBD are unknown (see Section 4.1).

Exon 11 of human *BRCA2* is large (4932 bp) (Figure 1b) and it contains eignt motifs, termed BRC repeats [48]. BRC motifs were initially described as non-identical 26 amino acid repeats [48] but were later defined as a 35 amino acid motif based upon a larger stretch of homology [39,40]. The BRC repeats directly bind the RAD51 recombinase [49,50], thereby leading to oligomerization of the protein on BRCA2. All vertebrates have eight BRC repeats arranged similarly within a central region of BRCA2 that spans more than 1000 amino acids, specifically 1002–2085 in human BRCA2 [48]. Interestingly, BRC repeats only represent ~25% of this vast region of BRCA2 and the intervening sequences are less conserved between species. It should be noted, however, that not all organisms have eight BRC repeats. For example, BRCA2 homologs in *U. maydis* and *C. elegans* have a single BRC repeat [51]. Relatively little is known about the structural requirements of the BRC repeat-containing element in BRCA2, including the need for all eight repeats, or for specific sequences and lengths of intervening regions.

A crystallographic study of the structure of a C-terminal fragment of BRCA2 bound to the DSS1 protein identified structural elements, present from a.a. 2482 to 3184, with predicted DNA-binding properties [41]. These elements, which lacked a recognizable sequence motif, include a helical domain and three oligonucleotide/oligosaccharide-binding (OB) folds. OB folds are found in most ssDNA-binding proteins [52,53], and residues within OB2 and OB3 directly contact and bind ssDNA [41]. In contrast, the distal end of a Tower structure within OB2 contains a three-helix bundle that appears to mediate binding to dsDNA [41]. Thus, BRCA2 contains a C-terminal DBD (C-DBD) that can bind both ssDNA and dsDNA. In this context, it should be noted that both ssDNA and dsDNA are present at DSBs following end resection of the DNA strands. Additionally, the crystal structure and a yeast two-hybrid study agree that DSS1 binds to the helical domain of the C-terminus of BRCA2, along with OB1 and OB2 [41,54]. 

The extreme C-terminus of BRCA2 has an additional RAD51-binding motif (a.a. 3270–3305) that does not have high homology to BRC repeats or intervening sequences [42]. Here, a direct interaction between BRCA2 and RAD51 is inhibited by cyclin-dependent kinase mediated phosphorylation of human BRCA2 at S3291 [42,55,56]. 

Based upon the presence of consensus motifs, human BRCA2 contains three putative nuclear localization signals (NLS) [43,57]. These elements are present in the extreme C-terminus of BRCA2 at a.a. 3263–3269 (NLS1), 3311–3317 (NLS2), and 3381–3385 (NLS3) on either side of the C-terminal RAD51-binding motif. While only NLS1 is well conserved between human, mouse, and rat BRCA2, each is highly enriched in positively charged residues, as is characteristic of NLS. 

Including the domains of human BRCA2 shown in Figure 1a, 2372 of the 3418 residues (~70%) are not associated with well-established structural or functional elements; the transactivation domain and N-terminal DBD are not included in this calculation because their physiological relevance is unclear. Thus, the functional role(s) of the vast majority of the BRCA2 protein is still unknown. Moreover, as discussed in Section 4, the functions of even known domains are not fully understood.

Regions of the human BRCA2 protein encoded by individual exons of *BRCA2* are shown in Figure 1b. This is important because several naturally existing alternatively spliced mRNAs, including those involving exons 3–7, skip particular exons and can potentially modify the pathogenicity of mutations, including truncating mutations, in *BRCA2* [34,58,59]. It is understandable that the in-frame deletion of exon 3 (∆ex3) transcript produces non-functional protein due to the absence of most of the PALB2-binding domain. In contrast, the ∆ex4–7 mRNA, with a deletion of 105 amino acids, encodes a DNA repair-proficient protein that is viable in mice, and which may ameliorate the phenotype of humans with loss-of-function (LOF) mutations in this part of *BRCA2* [34,58,59]. Notably, other in frame alternative splice forms exist, e.g., ∆ex10, ∆ex12, and ∆ex12–14, and might be important for germline mutation carriers who have nonfunctional mutations in these exons [34]. Thus, knowledge of the roles of different domains of BRCA2, which is largely lacking at present, will be critical for understanding the function of these splicing isoforms and their potential ability to rescue *BRCA2* deficiency.

## 3. Understanding the Roles of Different Domains within BRCA2, and an Overview of Models/Systems Developed/Utilized to Elucidate These Functions 

In the previous section, we established what is known about the domain structure of BRCA2. Here, we set the stage for characterizing the specific roles of known, as well as unknown, regions of BRCA2 in DNA damage responses (see Section 4). In particular, we explore why it is important to understand the function of the different domains of BRCA2 (Section 3.1) and examine the models/systems utilized to test BRCA2 function (Section 3.2). These approaches are key to further identifying still unknown functional domains of BRCA2, but also have limitations that may have constrained the definition of specific roles for known BRCA2 domains.

### 3.1. The Importance of Defining the Roles of Different Domains of BRCA2 for Understanding BRCA2 Function as a Tumor Suppressor and Harnessing Genetic Screens to Benefit Patients

While BRCA2 is broadly recognized to have various roles in DNA damage responses and the maintenance of genome stability [4,21], identifying the specific roles of domains throughout BRCA2 is a key to understanding its function as a tumor suppressor. Notably, only a small portion of BRCA2 has known functional domains. Thus, understanding BRCA2 function as a tumor suppressor will be aided by defining the individual role(s) of domains within unknown regions of the protein. Ultimately, since they may cooperate in the maintenance of genome stability, definition of the roles of regions throughout BRCA2 with distinct activities, may be necessary to fully understand BRCA2 function as a tumor suppressor. There is already evidence that different domains can have distinct activities. An example is the finding that the C-DBD and the C-terminal RAD51-binding domain are differentially required for HR and replication fork protection, respectively [14]. Elucidating the specific roles of different domains in BRCA2 may also indicate which activities are linked and which are independent. Further, although certain regions of BRCA2 could prove dispensable, the identification of structural elements, based upon sequence homology, might be aided by first defining new functional domains in the protein. Importantly, defining new domains in BRCA2 will also help with mapping regions of the protein that mediate any new functions of BRCA2 that might be identified in the future, potentially including those unrelated to DNA damage responses.

Mutations in *BRCA2* are associated with an increased risk for a variety of tumor types [16,17,18,19]. Thus, genetic screens are increasingly being used for the identification of patients and families with LOF germline alterations in *BRCA2* and related tumor suppressor genes. Once identified, healthy mutation carriers can undergo increased surveillance and/or prophylactic procedures to decrease the propensity for the manifestation of (a) tumor(s) [60,61]. However, the clinical significance of thousands of *BRCA2* variants, especially missense alterations, is unknown [37]. Therefore, these variants of uncertain significance (VUS) cannot provide any rationale for clinical decision making [37]. Importantly, while not currently used as a stand-alone method for determining the pathogenicity of variants [62], functional assays provide a greatly needed tool to aid in the classification of VUS of BRCA2 and related tumor suppressors [37,63]. 

Importantly, a profound understanding of the specific roles of various regions or domains of BRCA2 is needed to help design functional assays for characterization of VUS. First, such knowledge can be used for assay selection. For example, the C-terminal DNA binding domain (DBD) of BRCA2 is required for HR while other domains may not be involved in this process. Thus, different assays would be needed to functionally test missense or small insertions or deletions (indels) that are present in those domains. Second, certain regions or domains in BRCA2 may only be partially required for a particular function, meaning that inactivation of this domain will not lead to full LOF. Thus, functional assays utilizing mutants in which the entire domain is deleted can provide a baseline that represents a full loss of activity for that domain. Finally, missense alterations in domains without any known role in DNA damage responses might be accorded a lower priority for functional testing. VUS in these domains would seem less likely to affect BRCA2 functions related to its role as a tumor suppressor.

Genetic screens to detect deleterious mutations in *BRCA2* are also important as a basis for personalized/precision cancer treatments. BRCA2-deficient cells are sensitive to a range of established DNA damaging agents, many of which are used to treat cancer. This includes IR, camptothecin, cisplatin, and PARP inhibitors [6,7,8,9,10]. Thus, genetic screens to identify either germline or somatic *BRCA2* mutations in the tumor, which could sensitize a variety of tumor types to different therapeutic agents, hold promise for guiding the selection of tailored treatment options [37]. For example, the U.S. Food and Drug Administration has approved numerous PARP inhibitors for treating certain tumor types harboring either germline or somatic mutations in *BRCA1/2* [64,65]. 

Importantly, knowledge of the specific role(s) of particular domains can help exploit *BRCA2* deficiencies for therapeutic purposes. Ultimately, we hypothesize that different domains may have distinct roles in mediating cellular resistance to different DNA damaging agents. As such, knowing resistances to specific agents conferred by a particular domain are important for targeting mutations in that domain. To further illustrate this point, IR and cisplatin might be better used to target HR and replication fork protection defects, respectively, since one agent yields higher levels of DSBs, while the other yields more replication stress. Additionally, information on specific BRCA2 functions affected by mutations in a particular domain might lead to combinatorial treatments that better exploit the deficiency(s). For example, if a mutation affects HR but not the G2 checkpoint function, a DNA damaging agent might be effectively paired with a checkpoint inhibitor, such as an ATM or Chk1 inhibitor. Nevertheless, it should be kept in mind that truncating mutations, such as nonsense and frameshift alterations, may lead to low levels of the residual BRCA2 protein due to nonsense-mediated decay (NMD) and might thereby compromise all functions of BRCA2. Additionally, certain missense mutations and indels could lead to decreased stability of the resulting BRCA2 mutant, similarly compromising all of the various functions of the protein. In these cases, there would be no expected advantage to targeting domain-specific functions of BRCA2. 

### 3.2. Systems to Test BRCA2 Function Based upon the Expression of Mutants and Fragments of BRCA2, and Utilizing RNA Interference

The open reading frame of human *BRCA2* has ~10.3 kb and the protein has ~390 kD. Due, at least in part, to its large size, ectopic expression of vertebrate *BRCA2* for experimental purposes is difficult and challenging. Therefore, many experimental systems have been based upon disruption of the *BRCA2* gene, RNA interference (RNAi), or the testing of fragments (Section 3.2.1). In Section 3.2.2, we consider in greater depth the need for experiments based upon expressing mutants in the context of full-length BRCA2 and systems that have been utilized to achieve this. 

#### 3.2.1. Systems Based upon Gene Knockouts, RNAi, and the Use of Protein Fragments

One approach to studying BRCA2 function in DNA damage responses has been to test cells derived from mice with engineered disruption of the *Brca2* gene [2]. An important constraint presented by most *BRCA2* knockouts is the fact that *Brca2* is an essential gene, both in mice and cultured cells [66,67,68]. Thus, only hypomorphic mutations, such as deletion of exon 27 [2,68], may yield viable and proliferative cells, which are necessary for studies of DNA damage responses. Further, knockout alleles can result from frameshift mutations, leading to truncation and very low levels of the mutant protein due to NMD [68]. Another approach to the study of BRCA2 function involves RNA interference (RNAi) to deplete the protein [46,69,70], thus also leading to protein-wide perturbation of BRCA2. Notably, protein-wide disruption, which typically results from gene disruption or RNAi-based approaches, offers little or no insight into the function of specific domains of BRCA2.

An additional experimental approach has utilized small fragments of BRCA2, both for cell-free in vitro assays and in living cells [6,41,71,72,73]. For example, expression of single BRC repeats in cells interferes with BRCA2 function and thereby implicates the protein in different aspects of the DNA damage response [6,71,72]. In contrast, in vitro biochemical experiments using fragments of BRCA2 have been employed to ascertain the function(s) of particular domains [41,73] but are potentially inaccurate if cooperativity between multiple domains is required for that particular activity. Moreover, any activities identified in vitro may possibility be artefactual, again emphasizing the need for an expression system to confirm the findings in cells. 

Another strategy for improving the expression of *BRCA2* mutants has been to delete large regions that have not been previously identified as important for its function while retaining those believed to be most necessary or absolutely essential. Although low level expression of full-length BRCA2, which localizes normally to nuclei and displays enrichment in nuclear foci [74], can potentially be achieved utilizing a cDNA [49,72,74,75], deletion of large parts of BRCA2 results in a smaller “mini-protein” that can be expressed at higher levels [76,77,78,79,80]. Several studies have utilized miniaturized BRCA2 expressed in *E. coli*, insect, or human cells for in vitro biochemical experiments [77,78,79,80]. One such study fused the BRC3–4 repeats of human BRCA2 to the C-DBD [77]. The resulting peptide was 906 a.a. long, as compared to the much longer 3418 a.a. of full-length human BRCA2. Purified BRCA2 mini-proteins have, in vitro, many of the properties expected of full-length BRCA2, including the ability to bind RAD51 and DNA, promote nucleoprotein filament formation of RAD51 with ssDNA, and mediate recombination [77,78,79,80]. A mini-protein approach has also been used to test BRCA2 function in cells [76,78]. One of these studies demonstrated a role for multiple domains of BRCA2 in HR [76]. Other BRCA2 functions were not tested. Among the weaknesses of this approach, each of the mini-proteins in that study was presumably only partially active and the expression levels were not calibrated relative to full-length BRCA2 [76]. Because they lack certain regions of the protein, simple fragments, discussed above, and mini-proteins are not well suited to defining the function of the various domains of BRCA2. 

#### 3.2.2. Systems Based upon Ectopic Expression of Mutants of Full-Length BRCA2 

Multiple domains may potentially cooperate in mediating a particular function of BRCA2. Thus, for accuracy, the study of BRCA2 domains and their roles often requires the expression of variants/mutants of full-length BRCA2 in cells. Further, understanding BRCA2 function as a tumor suppressor demands experiments that utilize mutants of full-length BRCA2 to determine how various domain-specific activities are integrated together. Ultimately, such information, which is largely lacking at present, is required to make *BRCA2* deficiencies in tumors, identified via genetic screens, fully actionable.

Below, we discuss the various systems, based upon ectopic expression of mutants of full-length BRCA2, that have been utilized to test the cellular function(s) of its domains. In many cases, the approach was developed for an application other than defining and/or testing the roles of BRCA2 domains. Table 1 summarizes these approaches, along with expression of mini-proteins, the use(s) they were developed for, and notable limitations of each approach relevant to defining and testing functional domains in BRCA2.

In general, cDNA-based approaches have not yielded the robust, stable expression of full-length BRCA2 necessary to reliably dissect the function of BRCA2 domains. A notable exception is transfection of *Brca2*-deficient VC-8 hamster cells with human BRCA2 expression plasmids [1,36,81,82,83,84], often for the purpose of functional characterization of the clinical significance of missense BRCA2 variants [81,82,83,84]. Benign and pathogenic variants were, in control experiments, functional and non-functional, respectively. However, functional characterization of human BRCA2 VUS has largely been restricted to the C-DBD, where homology between human and hamster BRCA2 is the highest. Regions of inexact conservation occur throughout BRCA2 [37] and overall conservation of BRCA2 is relatively low [37]. For example, only 59% of residues in human and mouse BRCA2 are identically conserved [85]. Thus, while a cDNA-based approach is potentially rapid enough to elucidate the function of domains throughout BRCA2, heterologous expression systems may not be practical for this purpose due to concerns about homology. The case of the c.4146_4148del in-frame deletion variant (p.E1382del), which has a deletion within the BRC repeat region, is particularly noteworthy. Although expression of the human BRCA2-E1382del protein in VC-8 hamster cells was associated with full LOF for HR and hypersensitivity to MMC and IR [81], this variant was later demonstrated to be benign in humans [86]. This example clearly underlines concerns about the ability of heterologous expression, at least in hamster cells, to accurately test variants outside the C-DBD. 

Another heterologous system for the characterization of BRCA2 VUS is based upon rescue of lethality following conditional deletion of *Brca2* in mouse embryonic stem (ES) cells [67,87,88]. In this case, both human and mouse BRCA2 were capable of rescuing *Brca2*-deficiency in mouse ES cells [67]. For this assay, BRCA2 that contained variants was introduced into murine ES cells, which had floxed *Brca2* alleles, using a bacterial artificial chromosome (BAC) carrying the whole human *BRCA2* locus [67,87,88]. After floxing out the murine locus using the CRE recombinase, the effects of selected nonsense and missense variants of human BRCA2 on the assembly of Rad51 foci and HR, and on the maintenance of chromosome stability, were tested [67,87]. Again, assays were largely restricted to variants residing in more highly conserved domains, specifically the PALB2-binding domain and the C-DBD. In addition to concerns about inexact homology, this system is not rapid and may have difficulty testing essential domains for specific roles beyond rescue of lethality [34,88].

Recently, systems have begun to emerge for the expression of full-length human BRCA2 in human cells. One study characterized BRCA2 VUS by transfecting DLD1 cells, with an engineered *BRCA2* deficiency [92], using the piggyBac transposon system to introduce the BRCA2 cDNA into cells [89]. While having a system for testing variants and mutants of BRCA2 in human cells is desirable, the piggyBac transposon system is not ideal for defining domains in BRCA2 and elucidating their functions. In particular, the expression levels of full-length *BRCA2* were rather low and expression of the WT cDNA did not appear to fully rescue the *BRCA2* deficiency of the cells [89]. Additionally, the dynamic range associated with benign versus pathogenic variants of BRCA2 was much narrower than in other available systems. Thus, this system might not be sensitive enough to test the roles of domains that are only partially required for BRCA2 function. 

Full-length *BRCA2* has also been expressed in human cells, as well as yeast, using a BAC or phCMV for in vitro biochemical assays [1,90,93]. The levels of BRCA2 expression in human cells using a BAC may not be sufficient for cell-based assays. Further, it should be noted that yeast cannot be utilized for mapping functional domains in BRCA2 because of the absence of a BRCA2 homolog.

Another approach that can be used to test domains of BRCA2 is CRISPR (clustered regularly interspaced short palindromic repeats)-Cas9 mediated gene editing [94,95]. This system has been employed to create specific alterations of BRCA2 in human cells [91]. Because this approach is relatively labor intensive, this type of gene editing may not be suitable for generating the large number of mutations in different regions of BRCA2 that is required for detailed structure-function studies. Notably, while CRISPR-Cas9 has also been used recently for saturating mutagenesis of particular exons of *BRCA1* [96], to characterize variants identified in patients, such an approach has not yet been applied to *BRCA2*. This saturation mutagenesis approach is also not well adapted for mapping domains across BRCA2 because it must be developed on an exon-by-exon basis and does not generate specific deletions.

## 4. The Function of Different Domains of BRCA2 in DNA Damage Responses

Certain functions of BRCA2, including binding to interactors, such as PALB2, RAD51 and DSS1, and events related to HR, can be tested in vitro with biochemical approaches [1,38,39,78,79]. However, many molecular functions of BRCA2 in DNA damage responses can only be tested in living cells. In Section 4.1, we explore what is known about the function(s) of each established domain in BRCA2, beginning from its N-terminus, including examination of the role of particular domains in recruiting BRCA2 and RAD51 to sites of DNA damage. We further point out roles for particular domains in HR, G2 checkpoint regulation, replication fork protection, and cellular resistance to various types of DNA damage. Finally, we summarize what is known about how different domains may have antagonistic, cooperative, or redundant functions (Section 4.2). 

### 4.1. Domain-by-Domain Look at Roles of BRCA2 in DNA Damage Responses 

The PALB2-binding activity present at a.a. 21–39 of BRCA2 is involved in HR, as revealed by expressing fragments of *BRCA2*, full-length *BRCA2* [46,87], or using mutant mini-proteins [76]. Interestingly, cells derived from mice with a knock-in of a mutation known to abrogate the BRCA2–PALB2 interaction demonstrate a role for this domain in protecting stalled replication forks from nucleolytic degradation [97]. A role for the PALB2-binding domain of BRCA2 in preventing spontaneous and MMC-induced chromosome instability has also been suggested [87,97]. Additionally, a role for this N-terminal domain in mediating the proliferation of mouse cells was established using mutants of full-length human BRCA2 [87]. Further, in this same study, mutants of the PALB2-binding domain showed moderate sensitivity to various DNA damaging agents, including MMC, cisplatin and IR, as compared to the activity of WT BRCA2. A role for the PALB2-binding domain of BRCA2 in the recruitment of BRCA2 and RAD51 to sites of DNA damage, on the basis of their assembly into nuclear foci, has not been specifically tested. This might be inferred, however, by the fact that formation of both BRCA and RAD51 foci is defective in PALB2 mutants of the BRCA2-binding domain [98]. While not specifically tested, the PALB2-binding domain of BRCA2 might also be expected to have a role in mediating G2 checkpoint function, as both PALB2 and BRCA2 are individually required [13]. As an overview, in Table 2, we summarize the role(s) of the PALB2-binding domain, and other domains, in mediating specific functions of BRCA2 in DNA damage responses.

As noted earlier (Section 2), BRCA2 has a transactivating activity, present at a.a. 18–105, which is seen in the context of fusion to other factors with known transcriptional activity [45]. Interestingly, a benign variant of BRCA2, p.Y42C [99], which is devoid of transactivating activity [45], functions identically to wild-type (WT) BRCA2 with respect to cell growth and survival, either in the presence or absence of MMC or PARP inhibitors [67,81,89]. Further, p.Y42C does not compromise HR [81]. Thus, so far, all results suggest that the transactivating activity of BRCA2 has no role in cellular DNA damage responses. 

Another potential domain present in the N-terminus of BRCA2, the N-DBD at a.a. 250–500, has only been tested in cell-free systems in vitro [44]. Therefore, further examination, at a minimum expression of mutants of this region of BRCA2 in cells, is required before it can be considered to be a functional domain. 

Within a.a. 1002–2085 of BRCA2 are eight BRC repeats, each 35 amino acids long [39,40] that can directly interact with RAD51 [49,50]. Further, biochemical assays usng full-length protein have shown that BRCA2 can direct RAD51 to ssDNA, thereby stabilizing the filament RAD51 forms with ssDNA [1,90,93]. This work did not specifically attribute these activities to the BRC repeats, however. In vitro, individual BRC repeats can interact with either free RAD51 (BRC-1, 2, 3, and 4) or RAD51 oligomerized with ssDNA (BRC-5, 6, 7, and 8) with high affinity [39]. Interestingly, in vitro, BRC1–4 and BRC5–8 separately promote the strand exchange involved in HR when fused to the C-DBD better than the C-DBD alone, while BRC1–8 does not [78]. Further, consistent with the roles found for individual BRC repeats [39], BRC5–8 together had a greater affinity for the RAD51-ssDNA filament than BRC1–4 [78]. Thus, by having different RAD51-binding specificities, BRC1–4 and BRC5–8 may cooperate to promote nucleation and growth of the RAD51 nucleoprotein filament that initiates HR via strand invasion [39]. 

While the mechanistic role of the BRC repeats of BRCA2 in HR have been studied in vitro, as described above, knowledge of the function of BRC repeats in mediating cellular functions of BRCA2 is more limited. A mini-BRCA2 protein containing BRC1–4, BRC5–8 or BRC1–8, all fused to the C-DBD, was used to demonstrate that BRC5–8-DBD, but not BRC1–4-DBD or BRC1–8-DBD, promoted DSB-initiated HR in *BRCA2*-deficient DLD1 cells better than the C-DBD alone [78]. Levels of HR for BRC5–8 fused to C-DBD, measured utilizing a reporter assay, were similar to full-length BRCA2 [78]. Further, BRC5–8-DBD supported the assembly of RAD51 foci at levels intermediate between the C-DBD alone and full-length BRCA2. Moreover, fusions of BRC1–4, BRC5–8 and BRC1–8 to C-DBD conferred less cellular resistance to MMC than full-length BRCA2 but more than C-DBD alone, in both *BRCA2*-deficient VC-8 hamster and DLD1 human cells [78]. Other roles for the BRC repeats, such as mediating recruitment of BRCA2 to nuclear foci, cellular proliferation, G2 checkpoint function, replication fork protection and the maintenance of chromosome stability, were not tested [78]. Here, deletion mutants in the context of full-length BRCA2 are needed to clearly define the roles of the BRC repeats in DNA damage responses. This is especially important, as fragments of BRCA2, as well as BRCA2 mini-proteins, lack significant portions of BRCA2 and therefore cannot adequately account for cooperation between different domains. Finally, the cellular roles, if any, beyond “spacing”, for the regions between each BRC repeat are unknown at this time.

Biochemical studies, using full-length BRCA2 or fragments containing the C-DBD located between a.a. 2482–3184, suggest a role for this domain in mediating interactions of BRCA2 with ssDNA and in promoting recombination [1,41,90]. This specifically implicates OB2 and OB3 within the C-DBD, as they have been demonstrated to bind ssDNA [41]. Additionally, the helical domain and OB1 of the C-DBD are involved in binding DSS1, and BRCA2-DSS1 cooperate to mediate replacement of RPA with RAD51 on ssDNA [79,93]. However, as for the BRC repeats, the specific role(s) of the C-DBD in cells often can only be established definitively using mutants in the context of full-length BRCA2.

A role for the C-DBD in mediating DSB-initiated HR has also been revealed by cell-based assays conducted for the purpose of variant classification [81,82,83,84]. Many of these studies have employed heterologous expression of missense mutants of full-length human BRCA2 in *Brca2*-deficient VC-8 hamster cells [81,82,83,84]. In particular, pathogenic missense mutants in various portions of the C-DBD suggest that this domain is absolutely required for DSB-initiated HR, as levels associated with these missense mutations are similar or indistinguishable from that in cells reconstituted with the empty vector alone. Although truncating mutations are also defective for HR [81], missense variants are more informative about the specific role of the C-DBD; truncations of the C-DBD may result in undetectable protein due to NMD and cannot specifically test the role of the C-DBD, as other domains, such as the second RAD51-binding site and NLSs, are also eliminated. Studies based upon expression of missense mutants of human BRCA2 in *Brca2*-deficient mouse or hamster cells have also demonstrated a role for the C-DBD in certain other aspects of cellular DNA damage responses. This includes roles in cellular proliferation and in mediating resistance to MMC and IR [67,81,87]. A study based on a combined deletion of 9–10 amino acids of both OB2 and OB3 of endogenous *BRCA2* in chicken DT40 cells similarly found moderately increased sensitivity to DNA damaging agents, including IR, Olaparib, camptothecin, cisplatin, and MMC [8]. Interestingly, using this system, the C-DBD was found to be defective in supporting the assembly of both BRCA2 and RAD51 nuclear foci in response to IR [8]. Aside of deleterious missense variants having been identified in the helical domain and individual OB folds [63], there has been little dissection of the specific roles of each element of the C-DBD in DNA damage responses.

Closer to the C-terminus of BRCA2 is a second RAD51-binding motif/domain (a.a. 3270–3305; [42]) that interacts in vitro with RAD51 oligomerized into a filament with ssDNA and thereby protects the ssDNA from disassembly induced by BRC repeats [42,56]. It has been proposed that this RAD51-binding domain promotes nucleation of RAD51 filaments [56]. Notably, a residue within this RAD51-binding domain, p.S3291, is phosphorylated by cyclin-dependent kinases [55]. In vitro binding of the C-terminus of BRCA2 to the RAD51 nucleoprotein filament is inhibited when it is phosphorylated at S3291 [55]. Thus, phosphorylation of the C-terminal RAD51-binding motif at p.S3291 could act as a switch that turns off recombination, leading to binding of RAD51 predominantly by the BRC repeats encoded by exon 11 of BRCA2 and disassembly of RAD51 filaments [42,55]. Surprisingly, however, when the non-phosphorylatable p.S3291A BRCA2 mutant is expressed in the presence of full-length human BRCA2 in VC-8 hamster cells, the p.S3291A and WT BRCA2 proteins have similar activities in HR [14]. This suggests that the C-terminal RAD51-binding domain in BRCA2 is not involved in mediating HR. In contrast, HR is partially compromised when the p.S3291A mutant is expressed in the context of a BRCA2 mini-protein in these same *BRCA2*-deficient VC-8 cells [76]. Thus, it is paramount to realize that the results of functional assays may vary based on whether full-length BRCA2 or an artificial mini-protein is utilized. 

Another informative aspect of the BRCA2 p.S3291A case is that the mutant of full-length BRCA2 is still deficient in protection of replication forks from nucleolytic degradation in VC-8 cells treated with hydroxyurea (HU) [14]. Thus, this separation-of-function mutant demonstrates that HR and replication fork protection are independent activities/properties of BRCA2. In contrast, another functional unit in BRCA2, the PALB2 binding domain, mediates both HR and replication fork protection in the context of full-length BRCA2 [46,76,87,97]. Therefore, it is important to appreciate that the various domains in BRCA2 can have non-identical, domain-specific activities. As such, any understanding of BRCA2 function is incomplete without defining the role of each domain using the full-length protein. 

Based on expression of the p.S3291A mutant in VC-8 cells, the C-terminal RAD51-binding domain has a modest role in maintaining chromosome stability in the presence of HU [14]. Levels of chromosome instability associated with the p.S3291A mutant were intermediate to those observed in *BRCA2*-deficient VC-8 cells and their counterparts corrected with WT human BRCA2. Further, the C-terminal RAD51-binding domain was found to have no role in mediating cellular resistance to Olaparib. Other functions of the C-terminal RAD51-binding domain, including recruitment of BRCA2 and RAD51 to nuclear foci and regulation of the G2 checkpoint and of proliferation, are undetermined.

There are three putative NLS clustered close to the C-terminal RAD51-binding domain. Using GFP fused to full-length BRCA2 or small deletion mutants, one study determined that both NLS1 (a.a. 3263-3269) and NLS3 (a.a. 3381-3385) are necessary for the normal diffuse nuclear localization of the protein in the absence of exogenous DNA damage, but that NLS2 (a.a. 3311-3317) does not contribute [43]. Another study, which instead used a C-terminal fragment of BRCA2 fused to GFP, along with missense mutations of multiple residues in different NLS motifs, demonstrated that NLS1 and NLS2 are both required for full nuclear localization of BRCA2 but that NLS3 may not play any role [57]. Possible reasons for discrepancies may include utilization of full-length BRCA2 versus a fragment, as well as deletion of the putative NLS as compared to a missense mutagenesis strategy that could have resulted in ineffective abrogation of certain NLSs. Notably, role(s) for any of the putative NLSs in mediating specific BRCA2 functions in DNA damage responses are still undetermined.

The specific role(s) of known domains of BRCA2 in the DNA damage response have only been partially tested/identified (Table 2). It should be noted that the role(s) of all regions outside of these defined domains, including regions interstitial to the BRC repeats, are essentially unknown. There is evidence that these undefined regions are important to BRCA2 function, however. For example, a BRCA2 midi-protein that contains the undefined region of ~950 a.a. between the PALB2-binding domain and the beginning of the BRC repeats has a greater HR activity than BRCA2 mini-proteins that lack this region [76]. Since much remains to be learned about the roles of different domains throughout BRCA2, it will be important to define these functions in the future.

### 4.2. Functional Relationships between Domains of BRCA2 

Some domains may cooperate in mediating BRCA2 function in DNA damage responses. We hypothesize, for example, that the C-DBD may dictate the localization of the BRCA2-RAD51 complex to ssDNA at resected DSBs, while the BRC repeats regulate RAD51 assembly into a nucleoprotein filament that displaces RPA at these sites. Mutants of both domains might therefore be deficient for a particular function of BRCA2, such as HR, with each domain playing a different part. Defining specific roles of different domains within BRCA2 should be a key step in elucidating such cooperativity between domains. 

Based on the expression of mutants of BRCA2 mini-proteins in VC-8 hamster cells, the effects of deletion of the PALB2-binding domain on HR efficiency are more severe when the C-DBD is also mutated [76]. Thus, these domains may be partly redundant. One of the consequences of this is that mutation of a specific domain may not completely abrogate a particular function even if it has an important role. 

Using mini-BRCA2 proteins, the same study revealed, based on constructs with a complete deletion of the C-DBD, that this domain is not essential for HR [76]. However, as there was no comparison to the activity of full-length BRCA2 protein, it is possible that the C-DBD, while not required, still plays a role in HR in this system. This highlights the need to perform assays using mutants of full-length BRCA2 and with the WT (full-length) protein always present as a control.

It seems likely that the different domains in BRCA2 may, in part, have distinct activities that are integrated together into a BRCA2-dependent DNA damage response. For example, one domain might have a role in mediating HR while another promotes G2 checkpoint arrest in response to DSBs. Therefore, it is again apparent that an accurate understanding of BRCA2 function in DNA damage responses requires understanding the role(s) of each domain throughout the protein. 

## 5. Conclusions and an Outline of Parameters Necessary in an Ideal Expression System to Test the Function of Different Domains and Variants of BRCA2

Despite its well-established identity as a tumor suppressor, much remains to be understood about BRCA2. This includes elucidating functional domains in the majority of the protein that is uncharacterized and defining the exact role(s) of domains throughout BRCA2. This will help to comprehend how *BRCA2* functions as a tumor suppressor gene. Defining the roles of domains throughout BRCA2 will also help in harnessing the abundant potential of genetic screens to guide the selection of cancer prevention measures and therapeutic options in individuals that harbor sequence variants/mutations in *BRCA2.* In particular, this knowledge will help select functional assays for VUS that reside in particular domains of BRCA2. Additionally, knowledge of domain-specific activities may lead to novel strategies to exploit particular *BRCA2* deficiencies for therapeutic benefit.

BRCA2 is difficult to stably express in eukaryotic cells and this has clearly limited progress in understanding its function. For the purpose of domain characterization and performing functional tests of the effects of particular BRCA2 variants identified in patients, an expression system for full-length BRCA2 is needed that can overcome many of the limitations of existing systems. In our view, a system that is easy to use, and which is rapid enough for relatively high throughput testing of large series of mutants/variants located in different regions of the protein, is essential. Notably, gene editing systems may not be either rapid enough or suitable for testing mutants and variants located in different regions of the very large BRCA2 protein. Secondly, artificial mutants and patient-derived variants must be expressed in the context of full-length BRCA2, to distinguish no effect from a partial effect on BRCA2 function. Additionally, reliable testing of mutants and variants requires efficient expression at levels normal for endogenous WT BRCA2. Certain other systems, such as the piggyBac system, do not achieve suitable levels of ectopically expressed full-length BRCA2 in cells [89]. Further, the system should also be capable of expressing full-length BRCA2 in human cells. Unlike systems for the heterologous expression of human *BRCA2* in *BRCA2*-deficient rodent cells, systems for testing human BRCA2 in human cells are not limited by concerns and constraints related to inexact conservation of BRCA2 between species. Finally, protein-wide tests of domains of BRCA2 need to be done in the same cell type to best compare results based on mutation of different domains and to test the effects of distinct VUS that reside in them.

While a cDNA-based system could potentially meet many of the above criteria, especially for the characterization of patient-derived missense variants and indels, this system cannot reliably measure the effects of true splice variants on BRCA2 function. In such cases, patient-specific rescue transcripts, and different levels of NMD, influence the cellular response to DNA damage and replication stress [34]. Here, gene editing of the endogenous *BRCA2* gene by CRISPR-Cas9 or base-editor systems, or the use of BAC expression systems containing the whole human *BRCA2* locus seem to be better suited.

## Figures and Tables

**Table 1:** Approaches for testing the functions of specific domains in BRCA2, the purpose they were originally developed for, and key limitations for testing specific domains.

Approach	Purpose Developed for	Key Limitations	Refs.*
Expression of mini-BRCA2 proteins	Biochemical analysis of BRCA2 function Analysis of BRCA2 function in cells	Often lacks full-length comparison as a control	[77,78,79,80] [76,78]
Heterologous expression of cDNA in *BRCA2*-deficient hamster cells	Variant classification Other functional studies in cells	Inexact conservation between human and hamster BRCA2	[81,82,83,84] [1,36]
BAC expression in mouse embryonic stem cells with floxed *Brca2*	Variant classification	Inexact conservation between human and mouse BRCA2 Not very rapid	[67,87,88]
piggyBac transposon to transduce human cells with full-length BRCA2	Variant classification	Low level expression, including for WT BRCA2	[89]
phCMV or BAC for expression of full-length BRCA2 in human cells	In vitro biochemical assays	Levels of expression may not be sufficient for cell-based assays	[1,90]
CRISPR-Cas9 mediated gene editing of *BRCA2*	Engineering specific *BRCA2* mutations in human cells	May not be very rapid Not amenable to generating a protein-wide series of mutations	[91]

* If only one line of references is provided, this pertains to all information for that row; if two lines of references are given for a particular row, they are related to individual entries under “Purpose Developed for”.

**Table 2:** Roles of domains with known cellular functions in mediating specific BRCA2 functions in DNA damage responses.

Specific BRCA2 Function	PALB2-Binding Domain	BRC Repeats	C-Terminal DNA Binding Domain	C-Terminal RAD51-Binding Domain	NLS- 1, 2 and 3
BRCA2 Foci			+ [8]		
RAD51 Foci		+/− [78]	+ [8]		
HR	+/− [46,76,87]	+/− [78]	+ [8,81,82,83,84,87]	− [14,76]	
G2 Checkpoint					
Replication Fork Protection	+ [97]		− [14]	+ [14]	
Chromosome Stability	+/− [87,97]		+/− [87]	+/− [14]	
Proliferation	+ [87]		+ [87]		
Resistance to IR	+/− [87]		+ [8,81]		
Resistance to Chemotherapeutic Agents and/or MMC	+/− [87]	+/− [78]	+ [8,67,81,87]	− [14]	

+ indicates the domain is required for this particular function; +/− indicates a partial role, typically intermediate between the activity of WT BRCA2 and the empty vector; − indicates the domain was demonstrated to not have a role in this particular function; a blank box indicates that a particular function has not been directly tested for this domain.

## Data Availability

No new data were created or analyzed in this study. Thus, data sharing is not applicable to this article.
